# Characterization of the responses of the *caspase 2*, *3*, *6* and *8* genes to immune challenges and extracellular ATP stimulation in the Japanese flounder (*Paralichthys olivaceus*)

**DOI:** 10.1186/s12917-018-1763-y

**Published:** 2019-01-08

**Authors:** Shuo Li, Jiafang Li, Weijiao Peng, Gaixiang Hao, Jinsheng Sun

**Affiliations:** Tianjin Key Laboratory of Animal and Plant Resistance, College of Life Sciences, Tianjin Normal University, 393 West Binshui Road, Tianjin, 300387 Xiqing District China

**Keywords:** Extracellular ATP, Caspases, Immune response, Apoptosis, Teleost, *Paralichthys olivaceus*

## Abstract

**Background:**

Caspases are a family of conserved intracellular cysteine-dependent aspartate-specific cysteine proteases that play important roles in regulating cell death and inflammation. Our previous study revealed the importance of the inflammatory *caspase 1* gene in extracellular ATP-mediated immune signaling in Japanese flounder, *Paralichthys olivaceus*. To explore the potential roles of other caspases in *P. olivaceus* innate immunity*,* we extended our study by characterizing of the responses of four additional *P. olivaceus caspase* genes*,* termed *JfCaspase 2*, *3*, *6* and *8*, to inflammatory challenge and extracellular ATP stimulation.

**Results:**

Sequence analysis revealed that the domain structures of all the Japanese flounder caspase proteins are evolutionarily conserved. Quantitative real-time PCR analysis showed that the *JfCaspase 2*, *3*, *6* and *8* genes were expressed ubiquitously but at unequal levels in all examined Japanese flounder normal tissues. In addition, the basal gene expression levels of *JfCaspase 2*, *3*, *6* and *8* were higher than those of *Jf*C*aspase 1* in both Japanese flounder head kidney macrophages (HKMs) and peripheral blood leukocytes (PBLs). Furthermore, immune challenge experiments showed that the inflammatory stimuli LPS and poly(I:C) significantly modulated the expression of the *JfCaspase 2*, *3*, *6* and *8* genes in Japanese flounder immune cells. Finally, DNA fragmentation, associated with increased extracellular ATP-induced *JfCaspase 2, 3, 6* and *8* gene expression and enzymatic activity, was inhibited by the caspase inhibitor Z-VAD-FMK in the HKMs.

**Conclusion:**

Our findings demonstrate broad participation of multiple *caspase* genes in response to inflammatory stimulation in Japanese flounder immune cells and provide new evidence for the involvement of caspase(s) in extracellular ATP-induced apoptosis in fish.

## Background

Caspases are a family of conserved intracellular cysteinyl aspartate-specific proteases that trigger programmed cell death (apoptosis or pyroptosis) and inflammation to maintain homeostasis in organisms from worm to human beings [1–3]. The family of caspase proteins contains 14 members in mammals (caspases 1–3, 6–9, 12 and 14 in humans and mice; caspases 4, 5 and 10 in humans; and caspase 11 in mice). The major caspase proteins can be functionally classified into apoptotic caspases including the initiator caspases (caspases 2, 8, 9 and 10) and the effector/executioner caspases (caspases 3, 6 and 7), and the inflammatory caspases (caspases 1, 4, 5, 12 in humans and caspases 1, 11 and 12 in mice).

Caspases are tightly controlled as inactive zymogens (also known as procaspases) and are activated following signaling events promoting their cleavage and aggregation into dimers or macromolecular complexes [1]. The initiator caspases, which are activated by intrinsic and/or extrinsic signals, can cleave and activate the effector caspases, which in turn cleave target proteins during apoptosis, while the inflammatory caspases are primarily involved in pro-inflammatory cytokine processing during inflammatory processes [4] and in the promotion of innate immune responses to various internal and external stimuli [1]. Interestingly, both the initiator and effector functions are found within the inflammatory pyroptotic caspases (caspases 1, 4, 5 and 11) [2].

To date, several *caspase* genes have been cloned, including *caspase 1* from gilthead seabream, *Sparus aurata* [5]; *caspases 1*, *2*, *3*, and *9* from tongue sole, *Cynoglossus semilaevis* [6]; *caspases 1*, *2*, *3*, *8*, *9* and *10* from striped murrel, *Channa striatus* [7, 8]; *caspases 3*, *8* and *9* from sea bass, *Dicentrarchus labrax L.* [9–11]; *caspase 3* from rock bream, *Oplegnathus fasciatus* [12]; *caspase 6* from rainbow trout, *Oncorhynchus mykiss* [13]; *caspases 3* and *9* in large yellow croaker, *Pseudosciaena crocea* [14, 15]; and *caspases 1* and *10* from Japanese flounder, *Paralichthys olivaceus* [16, 17]. Previous studies have revealed the immunological significance of different caspases in fish. Upon overexpression of *caspases* in tongue sole, Long et al. found that *caspase genes 1*, *2*, *3* and *9* are essential to optimal defense against bacterial infection in fish [6]. Banerjee et al. reported that the caspase 3 protein mediated head kidney macrophage apoptosis during *Aeromonas hydrophila* infection in *Clarias batrachus* [18]. In addition, the involvement of the caspases 3 and 6 proteins in apoptotic cell death during red sea bream iridovirus infection has also been suggested [19].

Our previous studies revealed that extracellular ATP (eATP) is a potent signaling molecule in the activation of the innate immune responses in fish [20–22]. We recently identified and characterized a *caspase 1* gene (namely, *JfCaspase 1*) in Japanese flounder, *P. olivaceus*, and showed that eATP can rapidly upregulate *JfCaspase 1* gene expression and enhance its enzymatic activity in Japanese flounder immune cells, suggesting the involvement of caspases in eATP-mediated immune signaling in fish [17]. In this report, we identified and characterized the responses of four additional *caspase genes*, termed *JfCaspases 2*, *3*, *6* and *8*, to inflammatory stimulation in *P. olivaceus* immune cells. We also investigated the gene expression patterns and enzymatic activitiy induced by eATP stimuli. Our findings revealed that inflammatory stimuli, as well as the important danger-associated signaling molecule, eATP, have a broad effect on the gene expression of multiple *caspase* family members in Japanese flounder immune cells. In particular, we showed an association of eATP-induced DNA fragmentation with increased *JfCaspase 2*, *3*, *6* and *8* gene expression and enzymatic activity in Japanese flounder immune cells. Our findings suggest that caspase(s) may play an important role in eATP-induced apoptosis in fish.

## Methods

### Fish maintenance and tissue sampling

The experimental fish *P. olivaceus* were obtained from a local fish farm in Tianjin, China. Fish were maintained in an aerated running sea water system in the laboratory for two weeks before experiments. Only healthy fish without any pathological signs were selected for experimentation. To collect tissue, fish were euthanized with 0.25 g/L tricaine methanesulfonate (Sigma-Aldrich); blood were collected and tissues including the gill, head kidney, trunk kidney, heart, liver, skin, muscle, intestine and spleen were dissected from individual healthy Japanese flounder under sterilized conditions. Samples of the same kind of tissue from five individual fish were pooled, and total RNA was extracted (see below) to analyze the basal tissue expression of Japanese flounder *caspase* genes by quantitative real-time PCR (qRT-PCR).

### RNA extraction, cDNA preparation and gene cloning

Total RNA from cells and tissues was extracted using a PureLink® RNA Mini Kit and TRIzol reagent (Invitrogen), respectively, according to the manufacturer’s instructions. The integrity of the purified total RNA was examined with a 1.5% formaldehyde denaturing agarose gel. Then, the RNA was quantified by a NanoDrop spectrophotometer and treated with DNase I (Invitrogen, amplification grade) to remove genomic DNA contaminations following the protocol specified by the supplier. First-strand cDNA was then synthesized using a SuperScript III reverse transcriptase kit (Invitrogen) according to the manufacturer’s directions. The entire coding regions of the *JfCaspase 2*, *3*, *6* and *8* cDNA were amplified from Japanese flounder spleen or liver tissue using Platinum™ Taq DNA Polymerase (Invitrogen) with the primer pairs listed in Table 1, which were designed based on the available Japanese flounder *JfCaspase 2*, *3*, *6* and *8* cDNA sequences (GenBank accession numbers: XP_019948600.1, AFC60626.1, XP_019956800.1 and XP_019955218.1, respectively) in the GenBank database of the National Center for Biotechnology Information. The PCR products with the expected sizes were purified with a GeneJET Gel Extraction Kit (Thermo Fisher Scientific) and cloned into a pMD18-T vector (TaKaRa) for DNA sequencing.

**Table 1:** Primers used in this study

Primer name	Sequences (5’→ 3’)	Applications
JfCaspase 2-f	GGAGCAGCTGGACGATCGAC	Gene cloning
JfCaspase 2-r	ATATGTGCATGTGATCGATA	
JfCaspase 3-f	CAACAACAAGAACTTCGACAGG	
JfCaspase 3-r	TGTATATGTCAGGACAGTGCAA	
JfCaspase 6-f	CATTCGGAAGGAGAGAGGGA	
JfCaspase 6-r	GTGCTCAGCAACGACATACAG	
JfCaspase 8-f	CTGGCTTGTGTGGGAGGGAG	
JfCaspase 8-r	CAGCAATCTGTATCATACAGG	
qJfCaspase 2-f	CCTCGTGGTTTCGCCTTG	qRT-PCR
qJfCaspase 2-r	CGGTGGTCTGGTCGTTGG	
qJfCaspase 3-f	TCGCTGCAAATCGCTGGT	
qJfCaspase 3-r	CTGTGGAGAAGGCGTAGAGGA	
qJfCaspase 6-f	CTGAGCCACGGTGAGAACG	
qJfCaspase 6-r	ATTGTCCACGGCATCGCA	
qJfCaspase 8-f	CAGAGCCCTTCACGAGCAA	
qJfCaspase 8-r	CAAGGCACCGTCTCACCAT	
beta-actin-f	AGGTTCCGTTGTCCCG	
beta-actin-r	TGGTTCCTCCAGATAGCAC	

f and r denote forward and reverse primer, respectively

### Sequence data analyses

The obtained nucleotide and protein sequences were searched against the GenBank database using the BLAST program (http://www.ncbi.nlm.nih.gov/) to determine their sequence identities. The deduced amino acid sequences of the *JfCaspase 2*, *3*, *6* and *8* genes were translated using the ExPASy Translation Tool (http://www.expasy.org) and showed 100% sequence identity with their respective reference sequences in the NCBI database. Multiple sequence alignment was performed using the Clustal Omega program (https://www.ebi.ac.uk/Tools/msa/clustalo/). The protein domain structures were searched in the Conserved Domain Database (https://www.ncbi.nlm.nih.gov/cdd). The percentages of amino acid sequence identity were calculated by pair-wise alignments at the NCBI website with the default settings. A neighbor-joining phylogenetic tree was constructed based on the multiple sequence alignments using MEGA 5.0 software with the number of bootstrap trials set to 10,000.

### Preparation and cell culture of Japanese flounder head kidney macrophages and peripheral blood leukocytes

The procedure for the preparation of Japanese flounder primary head kidney cells was described previously [23]. Peripheral blood was collected from the caudal vein of Japanese flounder (average 800 ± 50 g) with a 10 ml heparinized syringe. The obtained primary head kidney cells and blood cells were then used to isolate the head kidney macrophages (HKMs) and peripheral blood leukocytes (PBLs), respectively, by discontinuous Percoll gradient centrifugation with a previously described protocol [24]. The viability of the isolated cells was examined by trypan blue exclusion assay. Then, the HKMs and PBLs were cultured in RPMI 1640 medium (Invitrogen) supplemented with 10% FBS and 1% penicillin-streptomycin liquid (Invitrogen) at 21 °C.

### Analysis of the basal expression levels of *JfCaspase* genes in Japanese flounder tissues and immune cells

The relative basal gene expression levels of *JfCaspase 2*, *3*, *6* and *8* in Japanese flounder normal tissues and immune cells were determined by qRT-PCR analysis. Briefly, Japanese flounder tissues, including blood, gill, head kidney, trunk kidney, heart, liver, skin, muscle, intestine and spleen, were collected from five healthy Japanese flounder (average weight 500 ± 20 g). Each kind of tissue was equally pooled to avoid individual variation, and the pooled samples were used for RNA purification. Total RNA was purified, digested with DNase I and transcribed into cDNA as previously described. The relative gene expression levels for the individual *JfCaspase* gene were determined by qRT-PCR (see below) with *beta-actin* serving as an internal reference gene.

### Immune challenge experiments

In vitro immune challenge experiments in Japanese flounder HKMs and PBLs with the pathogen-associated molecular pattern molecules LPS and poly(I:C) were detailed in a previous study [25]. Briefly, overnight-cultured Japanese flounder HKMs and PBLs (5 × 10^6^ cells/well) were treated with 20 μg/ml LPS or poly(I:C) (final concentration, dissolved in cell culture medium, Sigma-Aldrich) for 4, 8, 12, 24, 36 or 48 h. Cells without PAMP treatment served as the controls. After treatment, total RNA was purified and transcribed into cDNA, and LPS- and poly(I:C)-induced changes in *JfCaspase 2*, *3*, *6* and *8* gene expression were measured by qRT-PCR.

### Measurement of eATP-induced *JfCaspase* gene expression and enzymatic activity in Japanese flounder HKMs

To explore the responses of *JfCaspase* genes to eATP treatment, Japanese flounder HKMs (5 × 10^6^ cells/well) were stimulated with 1 mM ATP (dissolved in cell culture medium) for the indicated durations. eATP-induced *JfCaspase 2*, *3*, *6* and *8* gene expression changes compared with the expression in the untreated controls were measured by qRT-PCR.

The eATP-induced JfCaspase 2, 3, 6 and 8 enzymatic activity in the HKMs was determined with their respective enzymatic activity assay kits (Beyotime Institute of Biotechnology, China), according to the manufacturer’s directions. The eATP-induced enzymatic activity of the JfCaspase 2, 3, 6 and 8 proteins is presented as the change in their respective substrates (Ac-VDQQD-pNA, Ac-DEVD-pNA, Ac-VEID-pNA and Ac-IETD-pNA, respectively) into the yellow formazan product p-nitroaniline (pNA). Briefly, the HKMs were lysed on ice for 15 min followed by centrifugation at 15000 rpm for 15 min. The protein concentrations were determined with a Bradford protein assay kit (Pierce). The supernatant, containing equal amounts of total proteins (83.7 μg), was incubated in a 96-well plate with 30μl of the respective substrates at 37 °C for 2 h. The absorbance values of pNA released from the substrates in the clarified supernatant were measured at 405 nm (OD_405_) by a Tecan Infinite® M200 PRO multimode microplate reader (Switzerland). Each reaction was performed in triplicate. The eATP-induced JfCaspase enzymatic activity is presented as the pNA levels, which were calculated from a standard curve. The basal JfCaspase enzymatic activity in the HKMs without ATP treatment did not show any significant differences during the experiments (data not shown).

### DNA fragmentation assay

The Japanese flounder HKMs were treated with or without 1 mM ATP for 12 or 24 h in the presence or absence of pan-caspase inhibitor Z-VAD-FMK (Beyotime). After treatment, genomic DNA from the HKM cells was purified using a genomic DNA extraction kit for marine animals (Tiangen Biotech Co. Ltd., China) and treated with RNase A (Invitrogen). The same amount of DNA was loaded onto a 2% agarose gel, and DNA fragments were visualized by electrophoresis and staining with ethidium bromide. Without ATP treatment, no DNA fragmentation was observed in the HKMs during the experiments (data not shown).

### Real-time PCR analysis

The relative gene expression levels of the *JfCaspase 2*, *3*, *6* and *8* under normal conditions and different challenge conditions were determined by quantitative real-time PCR (qRT-PCR) analysis. qRT-PCR was performed on an Applied Biosystems® 7500 Fast Real-Time PCR System (Thermo Scientific) using an AceQ qPCR SYBR Green Master Mix kit (Vazyme Biotech Co. Ltd., China) according to the manufacturer’s recommendations. The primers for the detection of the individual *JfCaspase* genes are listed in Table 1. The amplification efficiencies of the primer pairs for qRT-PCR detection of the *JfCaspase 2*, *3*, *6* and *8* genes were 99.39, 99.14, 103.14 and 98.98%, respectively, which were determined based on the slopes of standard curves constructed for each amplicon using serial dilutions of the respective plasmid as templates. Gene expression levels were evaluated using the comparative 2^-△△Ct^ quantification method with *beta-actin* as an internal reference gene. Melting curve analysis and gel electrophoresis were performed at the end of amplification. The PCR products were further sequenced to ensure the specificity of amplification.

### Statistics

All data are presented as the mean ± standard deviation from triplicate experiments. The results were statistically analyzed with Student’s *t*-test for comparison between two groups. A *p* value less than 0.05 was considered statistically significant.

## Results and discussion

### Sequence analysis of Japanese flounder caspase proteins

The characteristics of the Japanese flounder caspase proteins are summarized in Table 2. *JfCaspase 1* encodes a 384-amino-acid protein with a predicted molecular mass of 43.7 kDa. *JfCaspase 2* encodes a 459-amino-acid protein with a predicted molecular mass of 52.09 kDa. *JfCaspase 3* encodes a 214-amino-acid protein with a predicted molecular mass of 23.92 kDa. *JfCaspase 6* encodes a 299-amino-acid protein with a predicted molecular mass of 33.70 kDa. *JfCaspase 8* encodes a 478-amino-acid protein with a predicted molecular mass of 54.33 kDa. In addition, the JfCaspase 1, 2, 3 and 6 proteins have similar isoelectric points that are close to 6, while JfCaspase 8 protein has a lower isoelectric point (Table 2).

**Table 2:** Characteristics of caspase proteins in Japanese flounder *Paralichthys olivaceus*

caspase	accession No.	pI	residues	Mw (kDa)	domain	predominant expression tissue
1	ARI72010.1	5.87	384	43.76	CARD,CASc	Skin [17]
2	XP_019948600.1	6.06	459	52.09	CARD, CASc	blood
3	AFC60626.1	6.09	214	23.92	CASc	skin, blood
6	XP_019956800.1	5.86	299	33.70	CASc	intestine
8	XP_019955218.1	5.13	478	54.33	DED, CASc	blood
10	BAE98150.1	5.36	494	55.87	DED, CASc	gills [16]

**Fig. 1:**
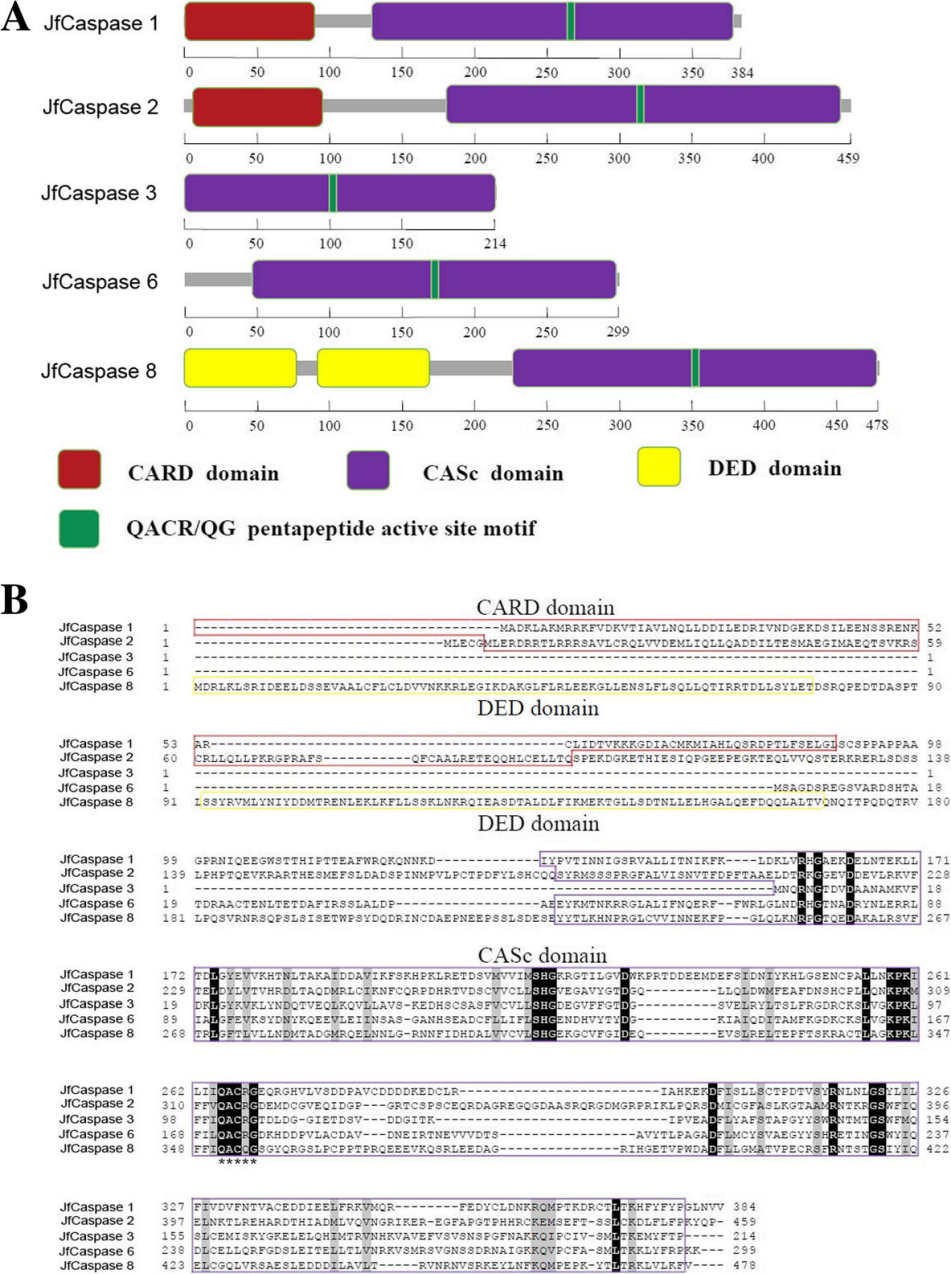
Domain organization and sequence alignment of Japanese flounder caspase proteins. **a** Schematic protein domain architecture of Japanese flounder caspase proteins. CARD: caspase recruitment domain; DED: death effector domain; CASc: caspase, interleukin-1 beta converting enzyme (ICE) homologues. **b** Multiple alignment of the amino acid sequences of Japanese flounder caspase proteins. The CARD domain, DED domain and CASc domains are boxed in red, yellow and purple, respectively. The conserved QACR/QG pentapeptide active site motif in all the JfCaspase proteins is denoted with stars. The numbers on the right-hand and left-hand sides are the numbers of amino acids in the JfCaspase proteins. The GenBank accession numbers of the caspase proteins from *Paralichths olivaceus* are ARI72010.1 (caspase 1), XP_019948600.1 (caspase 2), AFC60626.1 (caspase 3), XP_019956800.1 (caspase 6), and XP_019955218.1 (caspase 8)

Although the JfCaspase 1, 2, 3, 6 and 8 proteins exhibit different primary peptide sequences, they share a common caspase, interleukin-1 beta converting enzyme (ICE) homologues (CASc) domain containing a caspase active site pentapeptide (QACR/QG) motif (Fig. 1), a conservative feature of caspase-family proteins [26]. In addition, the JfCaspase 1 and 2 proteins contain an additional caspase recruitment domain (CARD), whereas JfCaspase 8 possesses two death effector domains (DED) required to signal apoptosis at the N-terminus, allowing these three proteins to complex with different adapter proteins and trigger different downstream signaling pathways. Furthermore, the JfCaspase 3 protein has an ^84^RGD^86^ motif that is involved in integrin recognition. These conserved features indicate that fish caspase proteins are structurally similar to mammalian caspase orthologs.

**Fig. 2:**
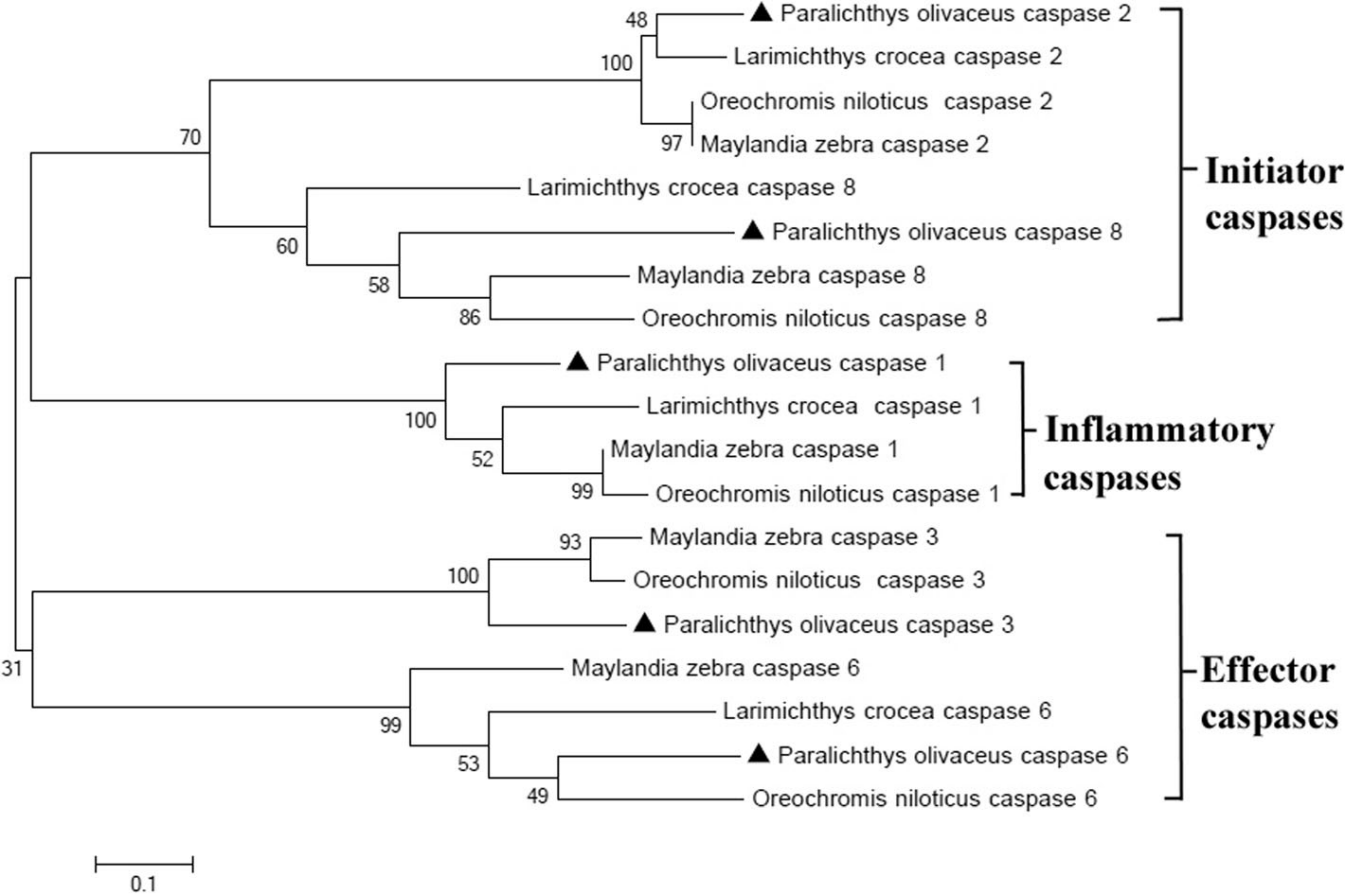
Phylogenetic relationships of Japanese flounder caspase proteins. Phylogenetic relationships among the Japanese flounder caspase 1, 2, 3, 6 and 8 proteins and their counterparts in other teleost species were determined using the ClustalW program**,** and the bootstrap-consensus neighbor-joining phylogenetic tree was built using MEGA software version 5.0 with the default parameters. The GenBank accession numbers for the caspases proteins from the different species are as follows: *Oreochromis niloticus* caspase 1: XP_005459436.1, caspase 2: XP_005455468.1, caspase 3: NP_001269823.1, caspase 6: XP_013131214.1, caspase 8: XP_003457507.2; *Maylandia zebra* caspase 1: XP_004543706.1, caspase 2: XP_004550818.1, caspase 3: XP_004549328.1, caspase 6: XP_014268841.1, caspase 8: XP_012778477.1; *Larimichthy crocea* caspase 1: KKF14496.1, caspase 2: KKF24100.1, caspase 6: KKF09831.1, caspase 8: KKF31210.1; *Takifugu ruubripes* caspase 1R: XP_003979716.1, caspase 2R: XP_011615364.1, caspase 3: AAM43816.1, caspase 6: XP_003972493.2; *Cynoglossus semilaevis* caspase 6: XP_008315389.1; and those in the list in the legend of Fig. 1

A BLAST search against the GenBank database revealed that the Japanese flounder caspase proteins share high sequence identity with their respective counterparts from other teleost species. Comparison of the amino acid sequences among the six Japanese flounder caspase proteins, however, revealed that they share less sequence identity (the maximum sequence identity is 21%, Table 3), indicating that they may have different structural features. Phylogenetic analysis revealed that the selected teleost caspase proteins are clustered into three subgroups: group 1 comprises the initiator caspase 2 and caspase 8 proteins; group 2 comprises the inflammatory caspase 1 proteins; group 3 comprises the effector caspase 3 and caspase 6 proteins (Fig. 2). This finding reflects their different structural relationships.

**Table 3:** Comparison of the amino acid sequence identities of caspase proteins in Japanese flounder *Paralichthys olivaceus*

caspase	Amino acid sequence identities (%)					
	1	2	3	6	8	10
1	100	13	11	10	11	10
2		100	11	10	11	13
3			100	21	13	11
6				100	15	13
8					100	18
10						100

### Co-expression of multiple *caspase* genes in Japanese flounder normal tissues and immune cells

**Fig. 3:**
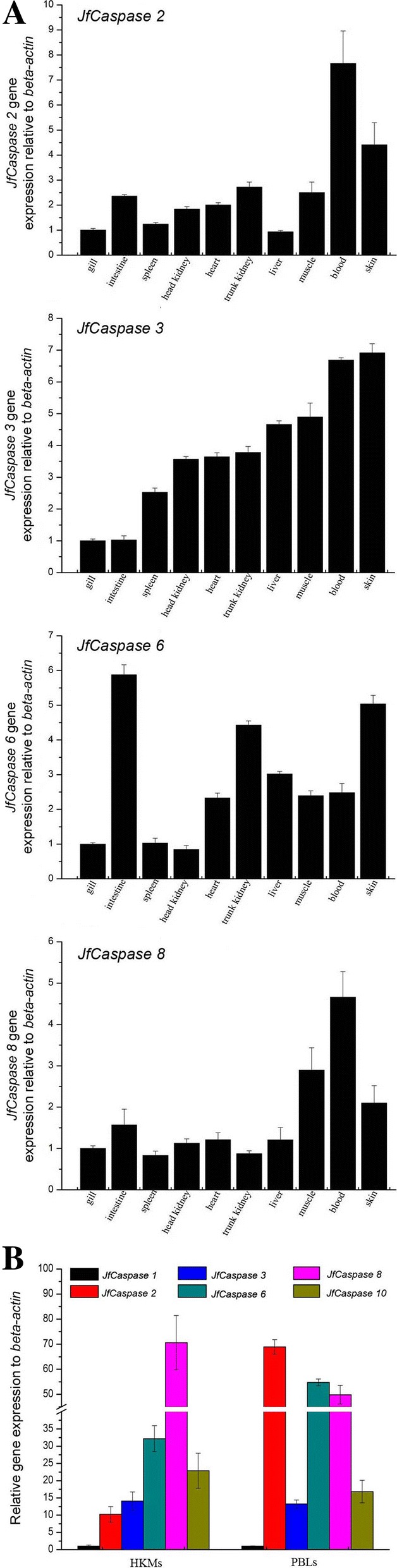
Expression of *JfCaspase* mRNA transcripts in Japanese flounder tissues and immune cells. **a** qRT-PCR analysis of the relative gene expression levels of *JfCaspase 1, 2, 3, 6* and *8* in Japanese flounder normal tissues. *Beta-actin* served as an internal reference gene. The error bars indicate the standard deviation from triplicate experiments. **b** qRT-PCR analysis of the relative gene expression levels of *JfCaspase 1, 2, 3, 6, 8* and *10* in Japanese flounder head kidney macrophages (HKMs) and peripheral blood leukocytes (PBLs), with *beta-actin* serving as an internal control gene

The expression of the *JfCaspase 2*, *3*, *6* and *8* mRNA transcripts in the Japanese flounder normal tissues are shown in Fig. 3a. *Caspase*-family genes are widely distributed in various tissues of various teleost species [7, 9, 10, 13, 15]. Similarly, all four *JfCaspase* genes are constitutively expressed in all examined Japanese flounder tissues but at unequal expression levels. This finding, together with our previous observation that the *JfCaspase 1* gene is widely present in Japanese flounder tissues [17], suggests that multiple *caspase* genes are required in the Japanese flounder body. However, the *JfCaspase 2* gene is expressed at the highest level in the blood, followed by the skin, trunk kidney, muscle, intestine, heart, head kidney, spleen, gill and liver. The *JfCaspase 3* gene is predominantly expressed in blood and skin and shows the lowest expression in the intestine and gill. The *JfCaspase 6* gene is primarily expressed in the intestine, with the lowest expression in the head kidney. The *JfCaspase 8* gene is highly expressed in blood but is expressed at lower levels in the spleen and trunk kidney. These observations indicate that different *caspase* genes are constitutively but unequally expressed in Japanese flounder tissues. Similarly, the predominant expression of the *caspase 2* gene in the blood of striped murrel, *Channa striatus* [7] and the abundant expression of the *caspase 3* gene in the blood of yellow croaker, *Pseudosciaena crocea* [15], have been reported previously. However, the *caspase 3* and *8* genes are mainly expressed in the spleen and liver, respectively, in *C. striatus* [7], and the *caspase2* and *3* genes are predominantly expressed in the muscle of tongue sole, *Cynoglossus semilaevis* [6]. These observations revealed that the dominant expression tissue of a given *caspase* gene varies among different teleost species, suggesting that *caspase* genes are diversely expressed in fish.

Next, qRT-PCR analysis was performed to compare the relative expression levels of *JfCaspase* genes in Japanese flounder immune cells. Figure 3b showed that the basal gene expression level of *JfCaspase 1* was lower than that of *JfCaspase 2*, *3*, *6*, *8* and *10* in both HKMs and PBLs. This lower expression may reflect a lower enzymatic activity of the JfCaspase 1 protein under resting conditions. The relatively higher gene expression levels of *JfCaspase 2*, *3*, *6*, *8* and *10* indicates that these genes are essential mediators of apoptosis in Japanese flounder immune cells. Alternatively, they may perform nonimmune functions under unstimulated conditions. For example, recent studies have indicated that caspase 2 activity is required for correct cell proliferation [27].

### Inflammatory stimulation-induced *JfCaspases* gene expression in Japanese flounder HKMs and PBLs

**Fig. 4:**
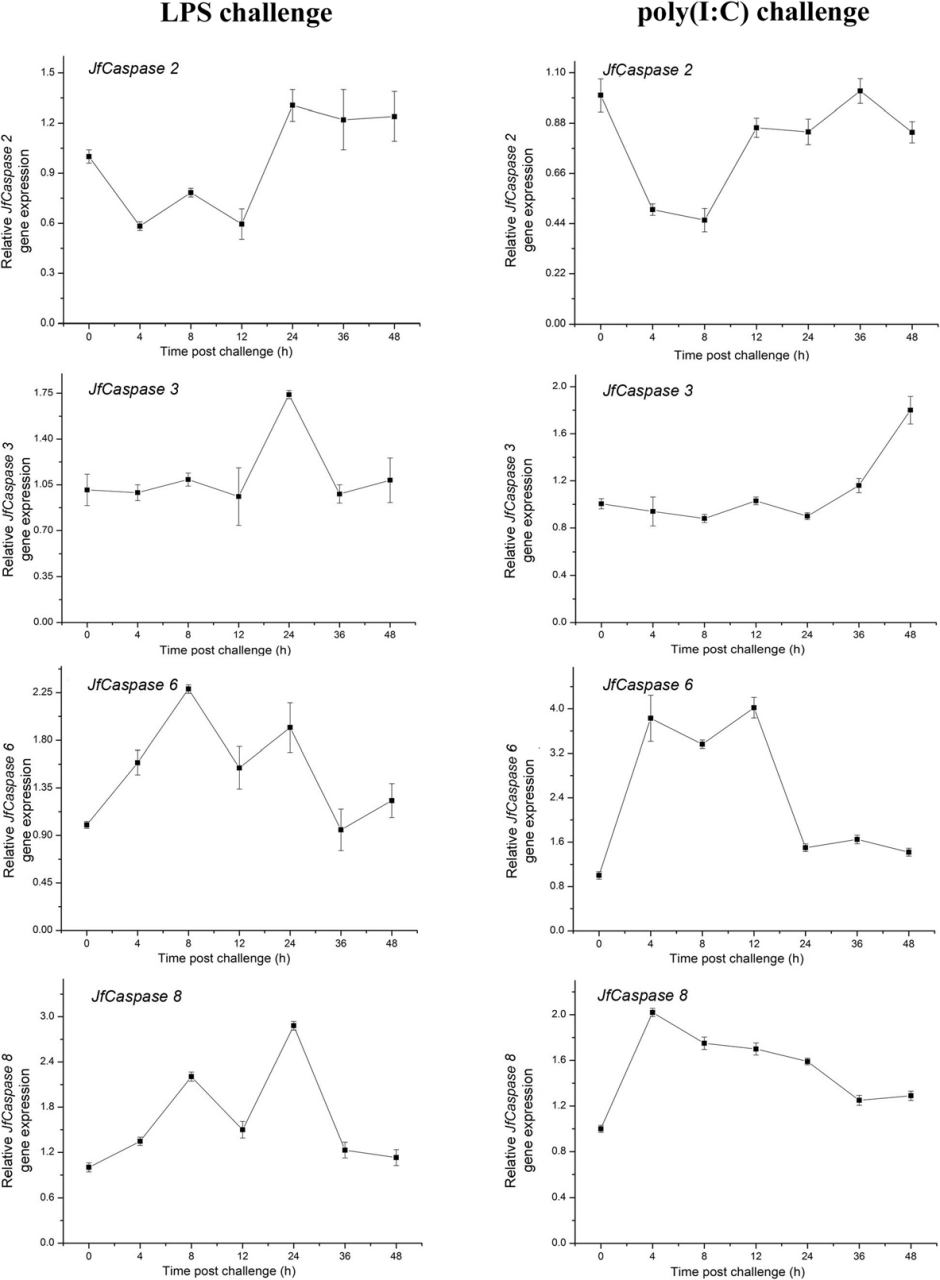
LPS- and poly(I:C)-induced *JfCaspase 1, 2, 3, 6* and *8* gene expression in Japanese flounder HKMs. Overnight-cultured Japanese flounder HKMs were stimulated with 20 μg/ml LPS (left column) or poly(I:C) (right column) for the indicated times. Total RNA from the cells was purified and transcribed into cDNA. *JfCaspase 1, 2, 3, 6* and *8* gene expression changes compared with the expression in the untreated controls were determined by qRT-PCR analysis. The values are presented as the means ± standard deviation (*n* = 3)

**Fig. 5:**
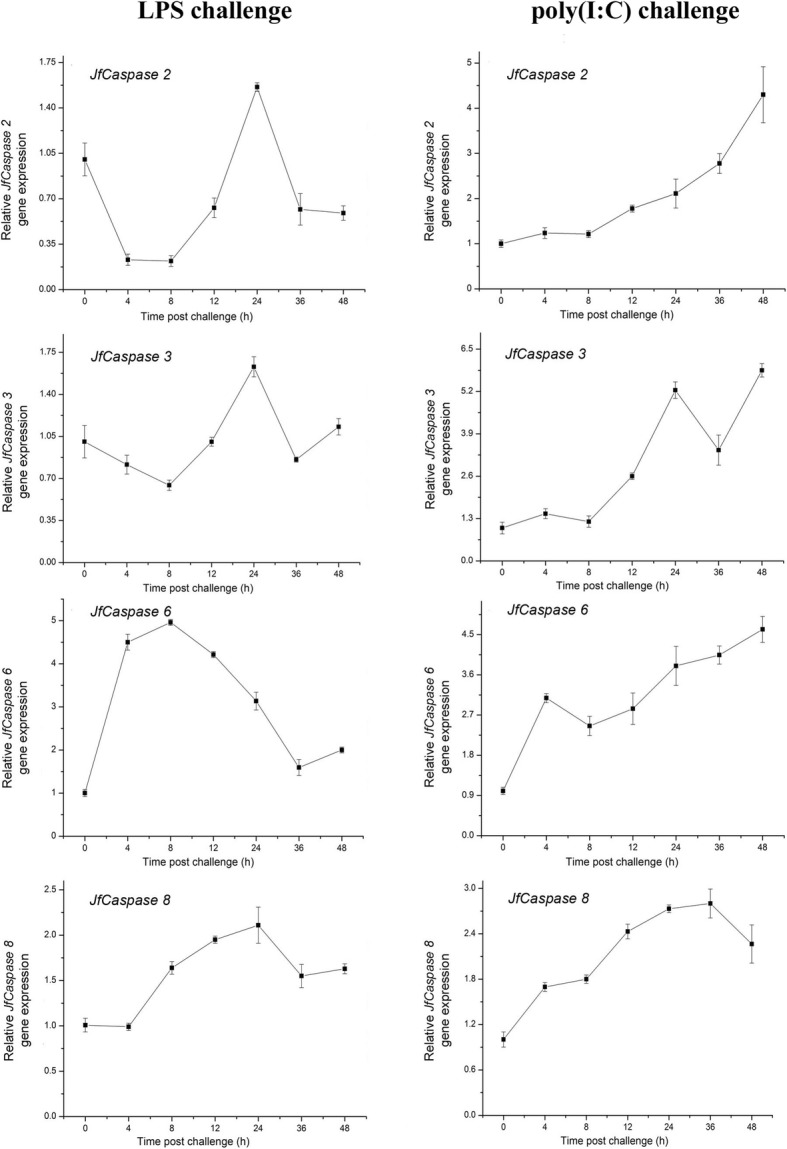
LPS- and poly(I:C)-induced *JfCaspase 1, 2, 3, 6* and *8* gene expression in Japanese flounder PBLs. Overnight-cultured Japanese flounder PBLs were stimulated with 20 μg/ml LPS (left column) or poly(I:C) (right column) for the indicated times. PAMP-induced changes in *JfCaspase 1, 2, 3, 6* and *8* gene expression were determined by qRT-PCR analysis. The values are presented as the means ± standard deviation (*n* = 3)

*Caspase* gene expression can be significantly modulated by different inflammatory stimuli. For example, the expression of the *caspase* genes *1*, *2*, *3* and *9* is modulated by bacterial infection in the tongue sole, *Cynoglossus semilaevis,* and has been suggested to play an essential role against bacterial infection [6]. Previous studies also reported the responses of caspase 3 and 6 proteins to viral infection in yellow-striped grunt (*Haemulon flavolineatum*) GF cells [19, 28] and the response of the *caspase 8* gene to bacterial infection in sea bass, *Dicentrarchus labrax L*. [9]. We therefore examined the effect of inflammatory challenges on *JfCaspase* gene expression in Japanese flounder immune cells. Figure 4 shows that the gene expression of the initiator *JfCaspase 2* was suppressed (by up to 0.6-fold), while the gene expression of the initiator *JfCaspase 8* were upregulated by LPS and poly(I:C) treatment (up to 2.8-fold and 2-fold higher than that of control group, respectively) in the HKMs. In addition, the gene expression of the effector *JfCaspase 3* in the HKMs was only upregulated approximately 1.8-fold at 24 and 48 h after LPS and poly(I:C) challenge, respectively. However, the gene expression of the effector *JfCaspase 6* was upregulated until 24 and 12 h after LPS and poly(I:C) stimulation (by up to 2.2-fold and 3.7-fold, respectively) in the HKMs, respectively. Again, in PBLs, the gene expression of the initiator *JfCaspase 2* gene expression was downregulated (up to approximately 0.2-fold lower than that of control group), while initiator *JfCaspase 8* gene expression was upregulated (up to approximately 2-fold higher than that of control group) at most time points after LPS stimulation. The gene expression of the effector *JfCaspase 3* was downregulated at some time points, while effector *JfCaspase 6* gene expression was significantly upregulated (up to approximately 5-fold higher than that of control cells) after LPS treatment in the PBLs (Fig. 5). These opposite expression patterns suggest that a compensatory mechanism may exist, i.e., when the expression of one *caspase* gene was suppressed, the other *caspase* gene was upregulated. Interestingly, all four Japanese flounder *caspase* genes were upregulated upon poly(I:C) stimulation in the PBLs (Fig. 5), suggesting that they may play an active role in response to viral infection. As gene expression changes may not always reflect the expression changes at the protein level, further investigations of the impact of PAMP stimulation on JfCaspase protein expression are needed. The basal gene expression levels of the *JfCaspase 2*, *3*, *6* and *8* genes in HKMs and PBLs without PAMP challenges did not show any significant differences during the experiments (data not shown). Taken together, our findings suggest that the *JfCaspase 2*, *3*, *6* and *8* genes may play a role in the response to bacterial and viral infections in the Japanese flounder, a hypothesis that needs to be clarified in the future.

### Extracellular ATP-induced JfCaspase gene expression, enzymatic activity and apoptosis in Japanese flounder head kidney macrophages

**Fig. 6:**
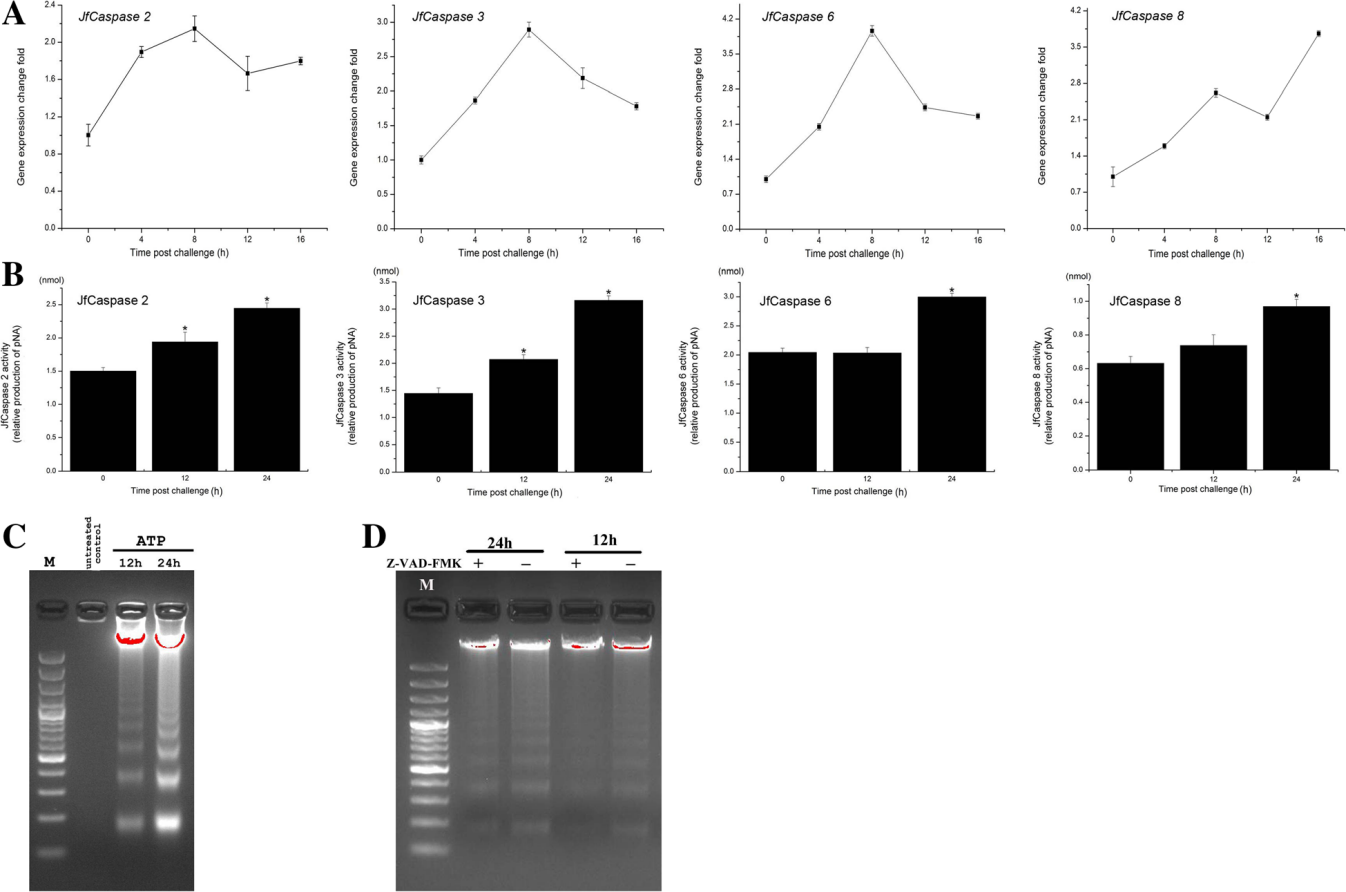
Extracellular ATP-induced changes in JfCaspase gene expression, enzymatic activity and apoptosis in Japanese flounder head kidney macrophages. **a** *JfCaspase 2, 3, 6* and *8* gene expression changes following 1 mM ATP stimulation in the HKMs were measured by qRT-PCR with *beta-actin* as an internal reference gene. **b** The eATP-induced changes in JfCaspase 2, 3, 6 and 8 enzymatic activity in Japanese flounder HKMs were measured with the respective enzyme substrates (Ac-VDQQD-pNA, Ac-DEVD-pNA, Ac-VEID-pNA and Ac-IETD-pNA, respectively), and are presented as the pNA levels calculated from a standard curve. Asterisks (*) indicate significant differences in JfCaspase activity compared with that of the untreated control group (*p < 0.05*). All data are presented as the means ± standard deviation of triplicate determinations from one representative experiment. The other two independent experiments showed similar results. **c** eATP-induced changes in DNA fragmentation in the HKMs. Untreated HKMs served as controls to show intact genomic DNA. In a parallel experiment, HKMs were treated with 1 mM ATP for 12 or 24 h. After that, genomic DNA was extracted, and DNA fragments were assessed by 2% agarose gel electrophoresis and ethidium bromide staining. M: 100 bp DNA ladder (Fermentas). **d** Inhibition of caspase enzymatic activity inhibited eATP-induced DNA fragmentation in HKMs. HKMs were pre-incubated with or without 100 μM pan-caspase inhibitor Z-VAD-FMK for 30 min and then treated with 1 mM ATP for 12 or 24 h in the presence or absence of 100 μM Z-VAD-FMK. DNA fragments were separated by 2% agarose gel electrophoresis and visualized with ethidium bromide staining

Caspase proteins can be activated by eATP [29]. For example, the activation of the caspase proteins 3 and 8 by eATP was observed in murine macrophages [30]. We previously showed that ATP was released from fish cells into the extracellular milieu during inflammatory stimulation and infection conditions via connexin 43 and or pannexin 1 channels [31–33]. Our previous studies also demonstrated that eATP is a potent signaling molecule in the activation of Japanese flounder innate immunity [20–22]. Specially, we showed that the Japanese flounder ATP-gated P2X7 receptor has an estimated EC_50_ of 743 ± 299 μM for ATP [20], and 1 mM ATP treatment can result in profound up-regulation of pro-inflammatory cytokine gene expression, iNOS activity and inflammatory mediators production, including ROS and NO, in Japanese flounder HKMs [22]. Recently, we found that eATP modulates *JfCaspase 1* gene expression and enzymatic activity in Japanese flounder immune cells [17]. To examine whether eATP stimulation can affect the expression of other *caspase* genes, the Japanese flounder HKMs were treated with 1 mM ATP, and the resultant gene expression changes are shown in Fig. 6a. Similar to *JfCaspase 1*, the *JfCaspase 2*, *3*, *6* and *8* genes were upregulated following ATP treatment although they exhibited different response patterns. Without ATP treatment, the basal gene expression level of *JfCaspase 2*, *3*, *6* and *8* did not show any significant differences during the experiments (data not shown). We next examined the enzymatic activity changes following eATP treatment. As shown in Fig. 6b, eATP treatment broadly upregulated the enzymatic activity of all the JfCaspase proteins in the HKMs. Specifically, the enzymatic activity of the JfCaspase 2 and 3 proteins was increased 12 h after ATP stimulation, while the increased enzymatic activity of the JfCaspase 6 and 8 proteins was observed 24 h after ATP treatment. The enhanced JfCaspase activity may reflect increased apoptosis activity in the HKMs. To test this hypothesis, we performed a DNA fragmentation assay. As shown in Fig. 6c, DNA fragmentation was observed following eATP treatment in the HKMs. However, this eATP-induced DNA fragmentation was inhibited by pre-incubation with the pan-caspase inhibitor Z-VAD-FMK (Fig. 6d). Previous studies have revealed that the *caspase 3*, *6* and *8* genes play important roles in apoptotic signal pathway in fish [9, 10, 13, 15, 18, 34, 35]. As eATP-induced apoptosis was reported to be mediated by activation of the Caspase 1, 3 and 8 proteins in murine macrophages [29, 30], the above observations thus highlight the important role of eATP in the induction of apoptosis through increases in the gene expression and enzymatic activity of multiple caspase protein family members expressed in Japanese flounder immune cells. Future studies are needed with specific inhibitors to inhibit the enzymatic activity of individual caspase proteins and determine the contributions of the individual caspase proteins to eATP-induced apoptosis in the HKMs.

## Conclusions

Our findings revealed the different responses of different *caspase* genes to inflammatory and eATP stimulation in Japanese flounder immune cells and provided new evidence for the involvement of caspase protein(s) in eATP-induced apoptosis in fish.

## Abbreviations

ATP: Adenosine triphosphate
CARD: Caspase recruitment domain
CASc: Caspase, interleukin-1 beta converting enzyme (ICE) homologues
DED: Death effector domain
eATP: Extracellular ATP
HKM: Head kidney macrophage
JfCaspase: Japanese flounder caspase
LPS: Lipopolysaccharides
PBL: Peripheral blood leukocyte
pNA: p-nitroaniline
poly(I:C): Polyinosinic-polycytidylic acid
